# Modelling response time in a mental rotation task by gender, physical activity, and task features

**DOI:** 10.1038/s41598-022-19054-2

**Published:** 2022-09-16

**Authors:** Patrick Fargier, Stéphane Champely, Raphael Massarelli, Laureine Ammary, Nady Hoyek

**Affiliations:** 1 University of Teacher Education, State of Vaud (HEPVD), UER EPS, 1014 Lausanne, Switzerland; 2 Inter-University Laboratory on Human Movement Biology (LIBM), Univ Lyon, University Claude Bernard Lyon 1, 69622 Villeurbanne, France; 3 Laboratory of Vulnerabilities and Innovation in Sport (L-Vis), Univ Lyon, University Claude Bernard Lyon 1, 69622 Villeurbanne, France; 4 Univ Lyon, University Claude Bernard Lyon 1, 69622 Villeurbanne, France

**Keywords:** Neuroscience, Psychology

## Abstract

Mental rotation (MR) is a spatial skill considered to be a key-component of intellectual ability. Studies have suggested that the response time (RT) in a MR task (MRt) might be influenced, with possible gender differences, by the practice of a physical activity (PA) and depending on the plane, direction, degrees of the MR and the frame of reference to perform it. The present study aimed at examining the respective influences of all these variables on the RT by developing a linear mixed-effect model from the RTs varying according to the MR plane, direction, degrees and frame of reference. The MRt was performed by 96 males and females, all undergraduate students, distributed in three groups (sedentary subjects, artistic gymnasts, and futsal players). The results showed that only gender had a main effect (faster log RT in males), probably task-dependent. The other variables interacted among them showing that: (a) the log RT may be influenced by rotations experienced during PA, in particular during the locomotion on a horizontal ground and (b) such influence mainly depends on the compatibility of the physical rotations experienced with the plane and the degrees of the MRt.

## Introduction

Mental rotation (MR) has been conceived as the ability to rotate the mental image of a two- or three-dimensional object^1^. The reality of MR had been supported by a study that examined the time required to identify whether two graphical images represented the same object, or a mirrored one^2^. The response time (RT) necessary to take a decision was found to be proportional to the angular difference of the depicted orientations of the objects, varying in the same plane from 0° to 180°. The results suggested that the subjects realized a MR to obtain a comparison^2^.

This aroused immediate interest in the field of cognitive science by contributing to the theoretical debate about the mental representation of information in humans, i.e.: whether any information is exclusively represented in a symbolic form or if it may be represented as mental images^3^. Different tasks and stimuli were also used to examine the assumptions supporting the concept of MR and/or to clarify the possible MR mechanisms^4–6^. These however, are not yet fully understood^7–9^.

It was also suggested that the measured performance of a MR task (MRt) may be a predictor of success in different educational programs (e.g.: anatomy learning^10^) or that it may be useful to assess competence in different professions (e.g.: air plane pilots^11^). In addition, some studies reported that males generally perform better than females when realizing a MRt^1,12–14^. Overall, the results contributed to put the emphasis on the possible determinants in the performance of a MRt, especially as it was shown that this performance may be improved by MR training^15–17^, notably by the practice of video-games involving spatial capacities^18–20^.

A possible link between physical activity (PA) and a MRt performance has been suggested^21^ since neuroimaging studies have shown that MR and movement preparation may share common functional cerebral mechanisms in the premotor cortex area^7,8^. Such possible link was supported by studies that compared experts in PAs that involve spatial capacities (e.g.: artistic gymnastics^22^, orienteering^23^, soccer^24^, springboard diving^25^) to non-athletes and it was confirmed by a meta-analysis^26^.

However, PA experts were not consistently found to prevail upon non-athletes. In some instances it is possible that the practised PA (e.g.: endurance running^23^) did not give a sufficient training in spatial capacities to facilitate the MR performance. It was also shown that, depending on the MRt, experts in a given PA may or may not perform better than non-athletes^24,27^, hence suggesting that the MRt performance might depend on both the practised PA and the performed MRt. This was supported by results obtained when comparing different PA experts among them (e.g.: gymnasts vs handballers^28^). The results raised the question on which “elements” might modulate the possible link between PA practice and the performance of a MRt.

The study of such “elements” has been approached by investigating the RTs performed by experts in artistic PAs (e.g.: artistic gymnastics, dance) and/or in team PAs (e.g.: handball, soccer). The results showed that the influence of a PA on RT might depend on the compatibility among the physical rotations experienced during the PA and some components of the MRt; in particular, the degrees of the rotation to be mentally realized (d°)^28,29^, the plane of rotation (Plane)^30^ and the direction of the rotation in the same plane (Dir)^31^.

The results of these studies also suggested that the MRts that encourage a motor strategy rather than a visual strategy (e.g.: imagining a self-body rotation vs visualizing a self-rotating stimulus^32^) might favour the realization of the task by using the spatial experience acquired during the PA, thus favouring the identification of oneself to the stimulus^33^. This might explain for example why gymnasts were found to be better performing than subjects who had no experience in self-body rotations during a MRt of left–right decision and when given an image of human body as stimulus; but only when such stimulus was presented in an upside-down orientation^29^.

Other studies not including PA experts suggested that a possible link between a common movement (e.g.: bipedal walk) and a MRt might also depend on the instructions given to perform the task by using an egocentric (Ego) frame of reference (FR), e.g.: imagining one’s own body rotation at the centre of an arrangement of objects, rather than by using an allocentric (Allo) one, e.g.: imagining the rotation of such objects around one’s own body^7,8,34–36^.

These reports however presented results obtained in separate experiments, with different subjects and different MRts, showing uneven effects of either, PA, d°, Plane, Dir and/or FR.

The present study investigated the respective influences of these same variables (PA, d°, Plane, Dir and FR) on the RTs obtained in a MRt performed by the same subjects while comparing males and females as a gender variable (Gender)^26^ and in the same experiment. The RTs obtained with this protocol have been interpreted with a powerful statistical model developed to obtain an accurate analysis of the results.

The study involved three groups of subjects each of them including young women and men of the same age span (see “Methods” section). The group of reference was constituted of sedentary subjects (without any regular physical activity; Sed) and the two others, of artistic gymnasts (Art) and of futsal players (Fut).

After familiarization with the experimental protocol, the subjects performed a MRt by identifying themselves with an avatar shown on the screen of a laptop and which was standing upright at the centre of a circle, on which four objects were arranged in opposition one to another. The subjects determined which object would be at a given place after rotation in one of 24 possible combinations of the following parameters: (a) d° (two conditions): rotation either at 90° or 180°, (b) Plane and Dir (PlaneDir; six conditions): either rotation in the horizontal Plane in right or left Dir; or in the frontal Plane in right or left Dir; or in the sagittal Plane in forward or backward Dir, and (c) FR (two conditions): either Allo or Ego. Each subject performed the MRt in each of these 24 conditions randomly.

The task performance was expressed as log RT (natural log) due to the RTs distribution highly skewed to the right. The log RTs obtained with the correct responses were modelled by using a linear mixed-effects model (LMEM)^37,38^. The model has been interpreted to clarify the possible main effects and/or interaction effects (fixed-effects) of the variables on log RT. This was done by taking into account the possible effects of the inter- and intra- individual variability (random effects) on log RT. As a complement, the error rates led to compute probabilities of success that were analysed by using a general linear mixed-effect model.

## Results

The measured performance of the MRt (expressed as log RT; untransformed mean RTs are shown in Supplementary Tables S1–S6 on line and the normal quantile plot of the residuals after log transformation is shown in Supplementary Fig. S1 on line) was statically modelled with LMEM by considering all order-one interactions. A synthetic representation of the model is given by the following Equation that shows the effects of Gender, PA, PlaneDir and FR (fixed-effects) on the performance of the MRt [expressed as log (RT)]. The asterisk (*) indicates the interaction between two variables. The term Subject (subject effect, *i.e.*: the inter-individual variation in log RT as a random effect) and the residual random error (intra-individual variation) followed non-correlated normal distributions with zero expectation.

$$\begin{aligned} {\text{log }}\left( {{\text{RT}}} \right) \, & = {\text{ Gender }} + {\text{ PA}}*{\text{d}}^\circ \, + {\text{ PlaneDir}}*{\text{PA }} + {\text{ PlaneDir}}*{\text{d}}^\circ \, + {\text{ PlaneDir}}*{\text{FR}} \\ & \quad + \, \left( {{\text{Subject random effect}};{\text{ intercept}}} \right) \, + \, \left( {\text{residual random error}} \right) \\ \end{aligned}$$

### Model relevance

The model (see the Equation) was statistically significant when compared to the null model including only one intercept term [likelihood ratio test: χ^2^(33) = 1122.7; *p* = 1.31e−214]. The corresponding percentage of explained variance was R^2^ = 42%.

A likelihood ratio test of the Subject effect (random effect due to inter-individual variations in log RT) showed the statistical usefulness of the term Subject in the equation, with χ^2^(1) = 359.9 and *p* = 2.96e−80. When measured in relative terms through the within-subjects correlation, the corresponding size effect was *ρ* = .22 (the standard deviations of the Subject effect and the residual random error also characterize this size effect in absolute terms with, respectively, *σ*_*s*_ = .19 and *σ*_*r*_ = .35).

A deviance analysis, performed to assess the importance of the various fixed-effects in the model described by the Equation, showed that only the main effect of Gender was of interest as it was the only variable found to influence the log RTs without interaction with any other variable. The deviance analysis showed that four interaction effects were statistically significant: PA*d°, PlaneDir*d°, PlaneDir*PA and PlaneDir*FR (in decreasing order of importance of their influence on log RT, see: Chi^sq^/df; Table 1).

**Table 1:** Analysis of deviance table (Type II tests) of main effects and interaction effects (order-one interactions; *) in the selected model of MR task performance (measured as the natural logarithm of the response time; log RT).

Fixed effects	Chi^sq^	df	*p*	Chi^sq^/df
**Main effects**				
d°	159.25	1	1.65e−36	159.25
PlaneDir	253.81	5	8.36e−53	50.76
PA	42.86	2	4.93e−10	21.42
Gender	14.47	1	1.42e−4	14.47
FR	2.04	1	.15	2.04
**Interaction effects**				
PA*d°	146.14	2	1.84e−32	73.07
PlaneDir*d°	52.63	5	4.01e−10	10.52
PlaneDir*PA	43.79	10	3.59e−6	4.38
PlaneDir*FR	20.81	5	8.81e−4	4.16

This analysis covered: d° (degrees of the rotation to be imagined), PlaneDir (plane and direction in which the rotation was performed), PA (physical activity of the subjects), Gender (sex of the subjects), FR (frame of reference used by the subjects to perform the MR task). The Table also shows, for each possible main effect and each statistically significant interaction effect, Chi-squared statistics (Chi^sq^), degrees of freedom (df), *p*-value (where *p* is the probability that a Chi-squared distribution with a given df is superior to the observed Chi^sq^) and a statistic (Chi^sq^/df) in order to stress the importance of the various effects (the higher the ratio, the more important the effect^39^).

### Gender main effect

Among the variables measured in this study, Gender was the only variable which significantly influenced the log RT while showing no statistically significant interaction with any other variable (Table 1). A significant difference in the means was found between females (Fe) and males (Ma) with: (Fe–Ma) = .16 ± .04 (Estimate ± standard error; SE); *z* = 3.80 and *p* = 1.42e−4. The percentage of variation (PV) in RT between two conditions can be computed from the corresponding difference in the means (ΔM), with PV = 100 × [exp(ΔM) − 1]. Therefore, when rounded to the nearest unit, PV indicated that the mean RT of the males was 17% faster than the mean RT of the females.

### Interaction effect between PA and d°

In the model described by the Equation, PA and d° had the most important interaction effect on log RT and this interaction was the only one that did not involve PlaneDir (see: Table 1). The interaction included two main effects showing that log RT was faster in the 180° condition than in the 90° condition and that the futsal group (Fut) was faster than the artistic gymnastics group (Art) while the sedentary group (Sed) was the slowest one (see: Table 2).

**Table 2:** Influence of d° (degrees of the rotation, i.e.: 90° or 180°) and PA (physical activity with three PA groups: sedentary subjects, Sed, artistic gymnasts, Art and futsal players, Fut) on the MR task performance (measured as log RT).

Difference	Estimate (log RT)	Standard error	*z-score*	*p* (> $\left| z \right|$)
**d°**				
90°–180°	.18	.01	12.40	< 2e−16
**PA**				
Sed–Fut	.33	.05	6.50	1.98e−10
Sed–Art	.19	.05	3.74	4.96e−4
Fut–Art	− .14	.05	− 2.76	.02

Multiple comparisons were carried out using the method of Bretz, Hothorn and Westfall^40^ (family-wise error rate set at 5%). The marginal means of the interaction effect between d° and PA on the log RT were used to compute the suitable contrasts in log RT. The corresponding differences (Estimate) are given as log RT values. Standard-error, *z*-score and *p*-value [single-step method; *p* > $\left| z \right|$] are also given.

**Figure 1:**
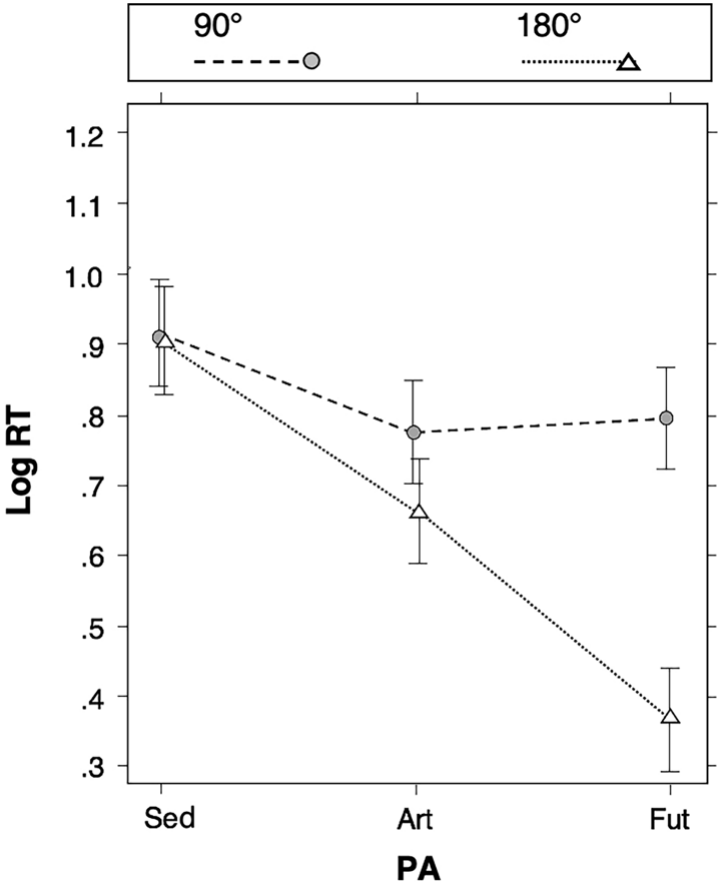
Effects of the interaction between PA and d° (PA*d°) on log RTs. The effect-plot^41^ shows the adjusted mean MR task performances (log RT) in each PA (physical activity) group (sedentary subjects, Sed, artistic gymnasts, Art and futsal players, Fut) according to the degrees of the rotation imagined (d°: 90° or 180°). The vertical line segments indicate ± 95% confidence interval. In each d° condition, broken lines connect the log RT values of the PA groups to emphasize the interaction (PA*d°).

Figure 1 shows the effects of the interaction between PA and d° on log RTs.

The comparisons between the mean log RTs obtained in the two d° conditions (90°–180°) showed no statistically significant difference (Estimate ± SE) in the Sed group [(90°–180°) = .01 ± .03; *z* = 0.37 and *p* = .975] (see: Supplementary Table S7 on line). On the other hand, a faster log RT in the 180° condition than in the 90° condition was found in the Art group [(90°–180°) = .11 ± .05; *z* = 4.38 and *p* = 3.53e−5] and, to a larger extent, in the Fut group [(90°–180°) = .43 ± .02; *z* = 16.92 and *p* < 1e−15] (see: Supplementary Table S7on line).

In the 90° condition, the multiple comparisons showed only one statistically significant difference among the PA groups with a log RT faster in the Art group than in the Sed group: (Sed–Art) = .14 ± .05, *z* = 2.58 and *p* = .044 (see: Supplementary Table S8 on line). In the 180° condition more clear-cut differences among PA groups showed that the Fut group was the fastest one and that the Art group was faster than the Sed one: (Sed–Fut) = .54 ± .05 with *z* = 10.02 and *p* < 1e−15; (Art-Fut) = .30 ± .05 with *z* = 5.57 and *p* = 7.21e−8; (Sed–Art) = .24 ± .05 with *z* = 4.47 and *p* = 5.73e−5 (see: Supplementary Table S8on line).

### Interaction effects with PlaneDir

**Figure 2:**
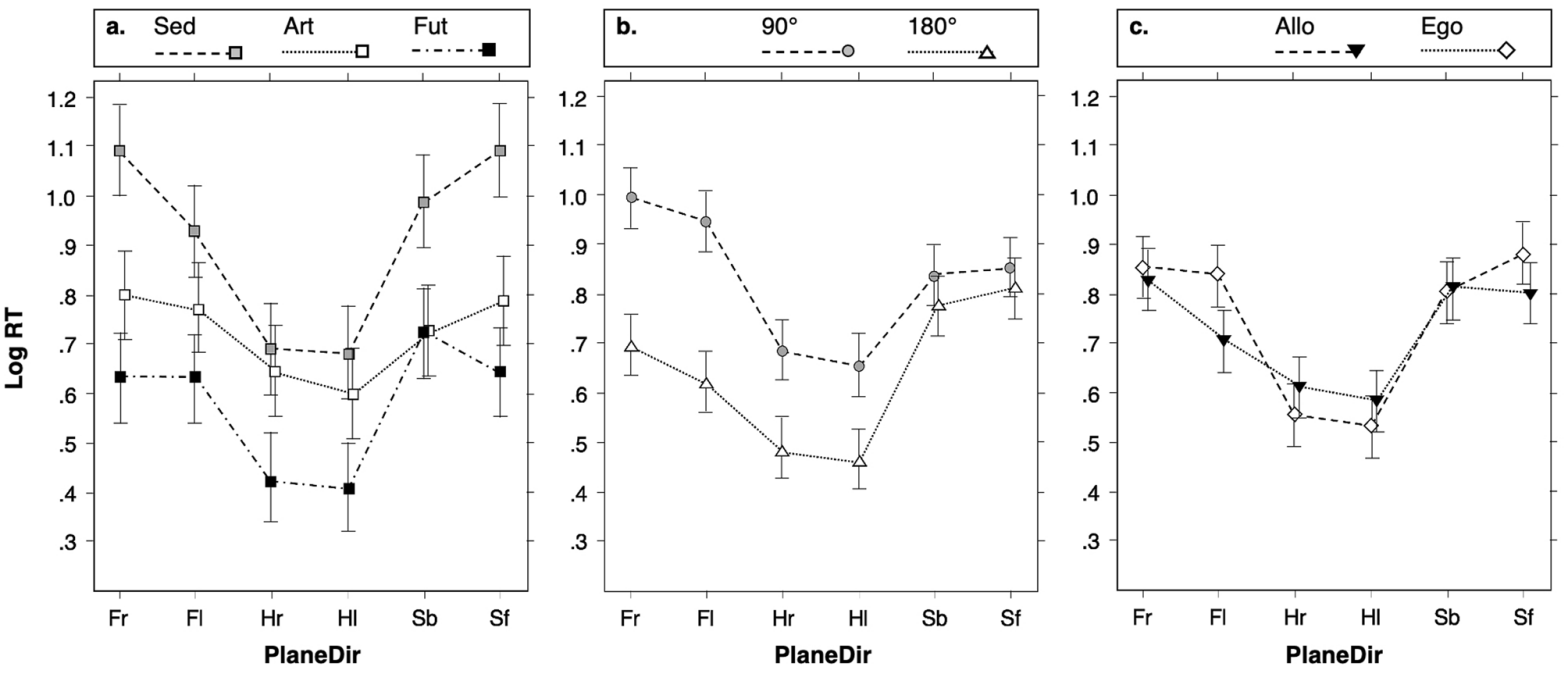
Interaction effects of PA, d° and FR with PlaneDir. The effect-plots^41^ a. b. and c. show the adjusted mean log RTs in each PlaneDir condition (plane and direction in which the rotation was imagined), i.e.: rotation in the horizontal plane on the right (Hr) or on the left (Hl), in the frontal plane on the right (Fr) or on the left (Fl), in the sagittal plane on a backward direction (Sb) or forward (Sf). In the effect-plots, the log RTs are shown according to: (**a**) the PA (physical activity) groups (sedentary subjects, Sed; artistic gymnasts, Art and Futsal players, Fut), (**b**) each d° condition (degrees of the imagined rotation: either 90° or 180°) and (**c**) FR conditions (frame of reference to imagine rotation), i.e.: either allocentric (Allo) or egocentric (Ego). In each effect-plot the vertical line segments indicate  ±  95% confidence interval. Broken lines connect the log RT values of each PA group (Fig. 2a), of each d° condition (Fig. 2b) and of each FR condition (Fig. 2c) to emphasize the interaction effects.

The effect-plots^41^ shown in Fig. 2 give a general overview of the effects of the interactions between PlaneDir and, respectively, PA, d° and FR.

#### Interaction between PlaneDir and PA

In each PA group (Fig. 2a; see also: Supplementary Table S9 on line) the mean log RTs in the frontal and sagittal planes were practically the same (the multiple comparisons showed no statistically significant difference, with *p* ≥ .979). The multiple comparisons also showed that in both the frontal and the sagittal planes the mean log RTs were slower than the mean log RT in the horizontal plane with *p* ≤ 3.15e−4.

From one plane to another the log RTs were more homogeneous in the Art group than in the Sed and Fut groups (Fig. 2a). The differences between the values of the mean log RTs in the horizontal plane and in both the frontal and sagittal planes (ΔMs; see: Supplementary information SI.4. on line) in the Art, Fut and Sed groups were: ΔM_Art_ = .15 ± .03 with *z* = 5.52 and *p* = 9.89e−8; ΔM_Fut_ = .25 ± .03 with *z* = 9.28 and *p* < 1e−15; ΔM_Sed_ = .34 ± .03 with *z* = 12.39 and *p* < 1e−15.

It was also found that the differences in log RTs between the two possible directions in each plane were generally moderate (Fig. 2a). The differences (three computed differences in each of the three PA groups; see: Supplementary Table S9 on line) only reached statistical significance in the Sed group, with log RT in the frontal (F) plane faster in the left (l) direction than in the right (r) direction with (Fr–Fl) = .16 ± .04, *z* = 3.62 and *p* = .005.

Moreover, in each plane, the log RT found in the Fut group was faster than in the Sed Group (Fig. 2a; see also: Supplementary Table S10 on line). The differences in the frontal, horizontal and sagittal planes were respectively: (Sed–Fut) = .37 ± .06 with *z* = 6.42 and *p* = 2.11e−9; (Sed–Fut) = .27 ± .06 with *z* = 4.74 and *p* = 3.82e−5; (Sed–Fut) = .36 ± .06 with *z* = 6.30 and *p* = 4.03e−9. In addition, the differences in log RTs between the Art and Sed groups (Fig. 2a; see also: Supplementary Table S10 on line) reached statistical significance in the frontal and sagittal planes with, respectively: (Sed–Art) = .22 ± .06 with *z* = 3.92 and *p* = .001 and (Sed-Art) = .29 ± .06 with *z* = 5 and *p* = 8.46e−6. The differences in log RTs between the Art and Fut groups only reached statistical significance in the horizontal plane (Fig. 2a; see also: Supplementary Table S10 on line): (Fut–Art) = − .21 ± .06 with *z* = − 3.65 and *p* = .004.

#### Interaction between PlaneDir and d°

In each d° condition (Fig. 2b; see also: Supplementary Table S11 on line), the multiple comparisons confirmed that the mean log RT found in the horizontal plane was faster than both mean log RTs in the frontal and in the sagittal planes, with systematically *p* ≤ 2.91e−11. In addition, the difference between the mean log RTs in the frontal and sagittal planes was found to vary depending on the d° condition (Fig. 2b; see also: Supplementary Table S11 on line). In the 90° condition, the log RT was faster in the sagittal (S) plane than in the frontal (F) plane with (F–S) = .12 ± .03, *z* = 4.60 and *p* = 5e−5. Conversely, in the 180° condition, the log RT was slower in the sagittal plane than in the frontal plane with (F–S) = − .14 ± .03, *z* = − 5.60 and *p* = 2.55e−7.

Whatever the d° condition, no statistically significant difference was found between the two possible directions in each plane, as shown by the Fig. 2b (three computed differences in each of the two d° conditions with *p* ≥ .352; see: Supplementary Table S11 on line).

In addition, no statistically significant difference between the log RTs in the two d° conditions was found in the sagittal plane (*p* = .237; see: Fig. 2b and Supplementary Table S12 on line) while the mean log RTs in the frontal and horizontal planes were faster in the 180° condition than in the 90° condition (Fig. 2b; see: Supplementary Table S12 on line); the respective differences were: (90°–180°) = .31 ± .03 with *z* = 12.18 and *p* < 1e−15, and (90°–180°) = .19 ± .03 with *z* = 7.71 and *p* = 7.44e−14.

#### Interaction between PlaneDir and FR

In each FR condition (Fig. 2c) the multiple comparisons showed that the mean log RTs in the frontal and sagittal planes were similar, with *p* ≥ .867 and slower than the mean log RT in the horizontal plane, with *p* ≤ 1.36e−10 (see: Supplementary Table S13on line).

With regards to the FR conditions (Fig. 2c) only one statistically significant difference in log RTs between the two possible directions in each plane was found. In Allo the log RT was faster in Fl than in Fr: (Fr–Fl) = .12 ± .04; z = 3.26 and *p* = .013 (see: Supplementary Table S13on line).

In addition (Fig. 2c), in each Plane condition no statistically significant difference in log RT between Allo and Ego was found (*p* ≥ .245; see: Supplementary Table S14on line) with the exception of the frontal plane. In this plane, the mean log RT was significantly faster in Allo than in Ego, but only in the left condition (Fl), with: (Allo–Ego) = − .13 ± .04, *z* = − 3.68 and *p* = .001 (see: Supplementary Table S14 on line).

Moreover, the mean log RT was faster in Ego than in Allo in the horizontal plane only (Fig. 2c), although the difference between the log RTs in Allo and Ego did not reach statistical significance (*p* = .245; see: Supplementary Table S14 on line). Interestingly the difference (Allo–Ego) varied significantly between the horizontal (H) and the frontal (F) planes [(Allo–Ego)_(H–F)_ = .13 ± .04 with *z* = 3.60 and *p* = .002; see: Supplementary Table S14 on line] and tended to vary between the horizontal and the sagittal (S) planes [(Allo–Ego)_(H–S)_ = .09 ± .04 with *z* = 2.39 and *p* = .092; see: Supplementary Table S14 on line].

### Probabilities of success

The obtained probabilities of success (Proba.) were from .87 to .98 (see: Supplementary information SI. 6., Tables S21, S24, S26, and S29 on line).

The statistically significant main effects and interaction effects, ranked in decreasing order by using the Chi^sq^/df criterion of McCullagh and Nelder^39^, were: PA*d° (Chi^sq^/df = 19.7; *p* < .001), PlaneDir (Chi^sq^/df = 18.8; *p* < .001), FR*d° (Chi^sq^/df = 11.8; *p* < .001), Gender (Chi^sq^/df = 11.2; *p* < .001).

For the effect-plots for each of these statistically significant effects, see: Supplementary Fig. S2 on line.

The multiple comparisons obtained in the two d° conditions showed that Proba. was higher in the 90° condition than in the 180° condition for the Sed subjects [90°–180° = .925 ± .149 (SE); z-ratio = 6.213; *p* < .0001] while the opposite was found in Fut (90°–180° = − .464 ± .165; z-ratio = − 2.817; *p* = .0049). In addition, Proba. in each of the 90° and 180° condition was significantly higher in Art than in Sed (with, respectively: (1) Art–Sed = 1.031 ± .239 with z-ratio = 4.311 and *p* < .0001, (2) Art–Sed = 1.725 ± .207 with z-ratio = 8.349 and *p* < .0001) and in Art than in Fut (with, respectively: (1) Art–Sed = 1.317 ± .232 with z-ratio = 5.676 and *p* < .0001, (2) Art–Sed = .622 ± .225 with z-ratio = 2.769 and *p* = .0155). No other comparison led to a result reaching statistical significance when considering PA*d° (see: Supplementary Tables S22 and S23 on line).

Considering PlaneDir, the results of the multiple comparisons showed that Proba.s in both Hl and Hr were significantly higher than Proba.s in Fl, Fr, Sb, and Sf (with *p* systematically < .0001; see: Supplementary Table S25 on line). No statistically significant difference was found between Hl and Hr (Hl–Hr = .292 ± .255; z-ratio = 1.145 and *p* = .863) and no other comparison led to a result reaching statistical significance when considering PlaneDir.

The multiple comparisons for FR*d° showed that Proba. was higher in the 90° condition than in the 180° condition in Allo (90°–180° = .571 ± .143; z-ratio = 3.989 and *p* = .0001) and that Proba. was higher in Ego than in Allo, in the 180° condition (Allo-Ego = − .620 ± .133; z-ratio = − 4.646 and *p* < .0001). No other comparison led to a result reaching statistical significance when considering FR*d° (see: Supplementary Table S28 on line).

Considering Gender, Proba. was found to be higher in Ma than in Fe (Fe–Ma = − .418 ± .125; z-ratio = − 3.344 and *p* = .0008; see: Supplementary Table S30 on line).

## Discussion

The present study examined in the same subjects and in the same experiment the respective influence of a series of between-subjects and within-subjects variables on the performance of a MR task (MRt), measured as log RT.

The log RTs were statistically modelled by using a linear mixed-effects model (LMEM), which showed that each variable had an influence on the log RT but only through one or more interaction(s) with another variable with the exception of Gender. This finding emphasizes the overall complex influence of all these variables on log RT, even at the level of order-one interactions only.

Gender was the unique variable found to influence the log RTs in all instances without interaction with any other variable (Table 1) and the main effect showed that the log RT was 17% faster in the male subjects than in the female subjects, which is consistent with the results obtained by studies using the Vandenberg and Kuse’s mental rotations test (VKtest)^42–44^, even if this classical test does not measure RTs.

Faster RTs were also found in males when compared to females^45^. Yet, when considering the error rates (ERs) gender differences may be more pronounced in some MRts^46^ (the ERs can be found in the Supplementary Table S15–S20 on line).

However, it has also been shown that the gender difference may be limited and even absent, depending on the stimuli and tasks^47,48^. Note that in the present study the stimuli were graphical images shown on the screen of a laptop. This might contribute to explain the male advantage in log RT found in the present study as it has been observed that 3D virtual reality, but not graphical images possibly abolish male superiority^49^.

The results also showed that Gender and PA may both influence the performance of a MRt without interaction between them, thus clarifying a point that had not been extensively studied^26^. In particular the interaction effect between PA and d° was the most important among all the interaction effects found in the present study (Table 1). This interaction showed: (a) no difference in log RTs between the 90° and the 180° conditions in the Sed subjects, and (b) faster log RTs in the 180° condition than in the 90° condition in the Art subjects and, to a larger extent, in the Fut subjects (Fig. 1). Several studies found a proportional relation between RT and the degrees of the rotation to be imagined in a MRt of same-different judgement between two stimuli^2,5,50^. Such proportional relation was not consistently found in other types of MRt (e.g.: left–right decision with a drawing of human body^50^), in particular when the MRt led sedentary subjects to imagine their own rotation at the centre of a display of objects, or the rotation of the objects around them^34,35^, as in the present study. In addition, the present study has shown clearly for the first time that both the Art and Fut subjects obtained faster log RTs in the 180° condition than in the 90° condition.

The literature shows that such absence of proportional relationship between RT and d° might be due to specific characteristics of some MRts^50^. A proportional relation between RT and d° has been classically found in MRts of same-different judgement between two stimuli because the individual MR, during these tasks, would be mainly constrained by the angular difference in the orientations of the stimuli. In other MRts that would instead favour the choice of a motor strategy and/or an egocentric perspective, MR would be also constrained by possibilities of the movements in human^50^. This might explain why a proportional relation between RT and d° has not been found in the present study as the MRt led to imagine oneself at the centre of an array of objects. However, this interpretation seems insufficient as the results showed no significant interaction between FR (Ego vs Allo) and d° but, instead, a significant interaction between PA and d°.

The Art subjects had obviously a larger experience of body rotation than the Sed subjects, which might explain the faster log RTs of the former compared to the latter in both the 90° and 180° conditions (Fig. 1). The acquired physical experience of the Art subjects is dependent on the informational and physical constraints of the motor tasks that they repeated during training^51^. This might explain why they obtained faster log RTs in the 180° condition than in the 90° one as: (a) their strict experience of a whole-body rotation at 90° (i.e.: the realization of a quarter-turn of the body) was mainly limited to the horizontal plane, while (b) they are trained to whole-body rotations at 180° in the horizontal plane but also in the frontal and sagittal planes, e.g., respectively: when realizing a handstand and during the performance of a cartwheel. The vertical (upright posture and the inverted one) may constitute a key-reference in the practice of different PAs^52^. Thus, in artistic gymnastics, both postures may be considered as key-positions to be reached in the frontal and the sagittal planes. Relative to the upright posture, the postures obtained in the frontal and sagittal planes after rotation at 0° and 180°, but not after 90°, might constitute key-references acquired by the Art subjects through training^52,53^. This might explain why the Art subjects were found to have faster log RTs in the 180° condition than in the 90° one.

The Fut subjects were found to have faster log RTs than the Art (and the Sed) in the 180° condition, but not in the 90° condition (Fig. 1). The futsal players must fetch information during the game very quickly, due to the relatively small playing area, and while the ball is moving in the three space dimensions concomitantly to moving partners and opponents. Such exchange between perception and action^54^ might thus increase bodily adaptation to rotations at more than 90° in the three space dimensions, even if futsal practice mainly requires rotations of the whole-body between 0° and 90°in the horizontal plane^55^. This might explain the faster log RTs obtained in the 180° condition by the Fut subjects. In addition, in the 180° condition (and not in the 90° condition), regardless the rotation plane or the used FR, the correct achievement of the task interchanged the positions of each pair of non-contiguous objects in our experimental conditions. The Fut subjects might have identified this key-recurrence, during the familiarization to the MRt, as futsal practice requires to develop a habit to find rapidly useful information for decision-making and action^56,57^ and/or to find opportunities to apply a learned procedure^58^.

The interaction effect between PlaneDir and PA showed more homogeneous log RTs from one plane to another in the Art group than in the Sed and in the Fut groups (Fig. 2a), a finding which is consistent with the results of a previous study with only male subjects performing a MRt of left–right decision^30^. Due to the specificities of the PA the Art subjects were probably more accustomed to body rotations in the frontal and sagittal planes than the Sed and the Fut subjects, while each of the Art, Sed and Fut subjects had a long-term experience of such rotations in the horizontal plane. This might explain why the differences in log RTs between the horizontal plane condition and each of the frontal plane and sagittal plane conditions were smaller in the Art group than in the other groups.

Another interesting finding was that in the 90° condition the log RT in the sagittal plane was faster than the one in the frontal plane, while in the 180° condition it was the opposite (Fig. 2b). This may be linked to usual experience of body rotations. Considering the 90° condition it should be noted that: (a) the possibilities to experience quarter-turns of the whole-body from a standing position in the frontal plane are rare, while (b) more opportunities exist to realize such rotations, at least quasi quarter-turns (90°) in the sagittal plane, e.g.: leaning. This might explain why, in the 90° condition, the log RT was faster in the sagittal plane than in the frontal plane. Moreover, depending on the FR condition, the MRt at 180° in the sagittal plane required the subjects to imagine either objects moving in their back (Allo FR) or their own bodies reaching an upside-down position (Ego FR). On the other hand the MRt at 180° in the frontal plane only had this second requirement, which might explain why the log RT at 180° was slower in the sagittal plane than in the frontal one. This might also explain why in the sagittal plane the log RTs in the 90° and 180° condition did not differ significantly, while in the frontal and horizontal planes the log RT was faster in the 180° condition than in the 90° condition.

On the overall, from one direction to the other in each plane, only moderate differences were found in the values of log RTs (Fig. 2) and such differences only reached statistical significance in two instances on a total of 21 (Fig. 2; Supplementary Tables S9–S14 on line). Both instances were found in the frontal plane with faster log RT in the left direction observed in the Sed group (Fig. 2a) and in Allo (Fig. 2c).The differences between Allo and Ego also reached statistical significance only in the left direction of the frontal plane with faster log RT in Allo (Fig. 2c). Such results, which may be linked to the complex influence of lateral preference, rotational preference, and/or previously experienced body rotations^59^, require further examination.

A more clear-cut phenomenon was instead observed when the three interaction effects involving PlaneDir were taken into consideration. In each PA group (Fig. 2a), in each d° condition (Fig. 2b) and in each FR condition (Fig. 2c) the log RT was faster in the horizontal plane than in both the frontal and sagittal planes. The performance of the MRt in the horizontal plane might generally be linked to the usual experience of rotation in the physical world, i.e.: body rotations and/or rotations of elements in the personal environment around the longitudinal axis of the body, when standing upright and on a horizontal ground.

This might explain why the Art subjects had faster log RTs in the horizontal plane than in both the frontal and sagittal planes while they had also more homogenous log RTs, from one plane to another, than the Sed and Fut subjects (Fig. 2a), mainly because the practice of artistic gymnastics may increase experience of body rotations in other planes than the horizontal one. The possible influence of body rotations experienced in everyday life is also consistent with the observation that the log RT tended to be faster in Ego than in Allo, but only in the horizontal plane (Fig. 2c). This might be anchored to the bipedal experience of horizontality in the physical world considering that such Ego advantage has been found only when the longitudinal axis of the imagined body was orthogonal to the MR plane^35^.

The analysis of the probabilities of success led to a series of complements.

A Gender main effect on log RT was found and, in addition, the complementary analysis of the probabilities of success (Proba.s) showed that Proba. was significantly higher in the Ma subjects than in the Fe subjects (respective Proba.s of .96 and .94; see: Supplementary Tables S29 and S30 on line). This confirmed that the Fe subjects had more difficulty than the Ma subjects to achieve the MRt accurately and as fast as possible under the present experimental conditions.

In addition, a significant interaction effect between PA and d° (PA*d°) was found both when considering the log RTs and when considering the Proba.s. When compared to the Sed subjects, the Art subjects were found to have faster log RT and higher Proba. in each of the 90° and 180° conditions (see: Supplementary Tables S22 and S23 on line). This confirmed that the Sed subjects had more difficulty than the Art subjects to perform the MRt in the two d° conditions. Interestingly, on the one hand, in the 90° condition, no difference in log RT between Art and Fut was found and, in the 180° condition, the Fut subjects were found to have faster log RT than the Art subjects. On the other hand, in both the 90° and 180° conditions, the Art subjects had significantly higher Proba.s than the Fut subjects (see: Supplementary Tables S21, S22, and S23 on line). This suggests that the Fut subjects, when compared to the Art subjects, performed some items of the MRt quickly to the detriment of accuracy in the given responses.

Moreover, no difference in log RT between the 90° and 180° conditions was found in the Sed subjects while these subjects had higher Proba. in the 90° condition than in the 180° one. This suggests that, in the 180° condition, when compared to the 90° condition, the Sed subjects possibly performed the MRt quickly to the relative detriment of accuracy. Such possibility was not shown by the Art subjects who had faster log RT in the 180° condition than in the 90° condition with Proba. that did not significantly differ from one d° condition to the other, and in the Fut subjects who obtained faster log RTs and higher Proba. in the 180° condition than in the 90° condition.

Overall, the study of the log RTs obtained with correct responses and the complementary study of the Proba.s showed possible speed-accuracy trade-offs, which were not general (e.g.: in the 90° condition, the global Proba. was 0.98 in the Art subjects and 0.93 in the Fut subjects, and in the 180° d° condition, the global Proba. was 0.97 in the Art subjects and 0.95 in the Fut subjects; see: Supplementary Table S21 on line), but this will require further examination. In particular, this might contribute to determine whether, and to what extent, such possible speed-accuracy trade-offs might depend on the practiced PA.

Considering FR, the results confirmed the complex influence of the within-subjects variables on the MRt performance. The study of the log RTs showed a significant interaction between FR and PlaneDir and the study of the Proba.s, a significant interaction between FR and d°. On the one hand, log RT in Allo was faster in Fl than in Fr and log RT in Fl was faster in Allo than in Ego. On the other hand, Proba. in Allo was higher in the 90° condition than in the 180° condition and Proba. in the 180° condition was higher in Ego than in Allo. This suggests again possible influence of lateral preference, rotational preference and/or experiences in body rotations^59^ that might lead to specific investigations.

Finally, the study of the log RTs exhibited three significant interaction effects (PlaneDir*PA, PlaneDir*d°, and PlaneDir*FR) leading to underline that the log RT was faster in the horizontal plane than in both the frontal and sagittal planes. The Proba.s were also found to be higher in the horizontal plane than in both the frontal and sagittal planes. This thus confirmed that the subjects had more difficulty to perform the MRt both quickly and accurately in the frontal and sagittal planes than in the horizontal plane. This suggests again possible influence of the usual experience of rotations when standing upright on a horizontal ground.

On the whole, the results of the present exploratory study show the interest of taking into consideration Gender, PA, d°, PlaneDir and FR to study the RTs obtained in a MRt at the same time and in the same experiment. In particular, this allows to approach the complex influence of such between-subjects and within-subjects variables on RT. Naturally this does not exclude that further research should be done to approach more in-depth this complex influence. Additional variables regarding the level of expertise in a given PA and/or the fitness level might notably help to clarify possible link(s) between physical activity and the performance of a MRt. Other types of MRts might also be used (e.g.: MRts of same-different judgement between two stimuli, MRts of left–right judgement with a single stimulus). Such an approach might notably yield a more in-depth knowledge on gender differences, which might be more or less accentuated depending on the MRt type^46^.

In addition, the present study also showed the interest of a complementary study of the error rates taking into consideration these same between-subjects and within-subjects variables at the same time and in the same experiment. In particular, this suggested the interest of studying more specifically the peculiar conditions that would lead to a speed-accuracy trade-off depending on the physical activity regularly practiced. This also suggested the interest to further examine the influence of FR on both log RT and Proba.

Overall, the present study showed that such approach of RT and ER is valuable to clarify the complex relations between the various determinants of the performance of a MRt. More generally, it might be interesting to consider order-two and/or order-three interaction levels, but with a much larger number of experimental subjects.

In conclusion, the present results show that only the gender variable had a main effect on the log RTs and that the faster log RT observed in males may be possibly linked to features of the performed MR task. Interestingly, the other variables were only involved when studied as interaction effects on log RT. These interaction effects suggested that the log RTs might be influenced by the PA performed usually by the subjects, depending on the rotations usually experienced during PA and on the compatibility between the experienced rotations, the degrees and/or the plane of the rotations to be mentally done. More generally, the results suggest that such complex influences might depend on the biomechanical possibilities, gravity-dependent and anatomically constrained that humans have in experiencing rotation movements and rotations of objects in the physical world. Finally and concerning the debate about the possible types of informational representation in humans^3^, our results may suggest the possibility of conceptual images rooted in the bodily experience.

## Methods

The study was approved by the ethical board for research of Claude Bernard University Lyon 1. In agreement with the Declaration of Helsinki (2013), the subjects were informed about the aim of the study and of the main lines of the experimental procedures, but they were left uninformed of possible scientific results of the experimental variables. The subjects participating to the experimental protocol gave their written informed consent and the experiment was performed in accordance with relevant guidelines and regulations.

### Subjects

A total of 96 undergraduate students (47 males and 49 females) took part to the experiment. All the subjects were right-handed and had normal or corrected-to-normal vision. They were distributed in three groups: (a) sedentary subjects (Sed, as a reference group), (b) artistic gymnasts (Art) and (c) futsal players (Fut). The Sed subjects had no regular physical activity while the Art and Fut subjects were regularly engaged in the practice of their respective PA of acquaintance. The subjects practiced their respective PA in university competitions at a regional level and had followed training in their respective PA three times per week for two years at least. The Sed, Art and Fut groups were of the same age span, and respectively included: (a) 31 subjects aged of 18.74 ± .77 years (mean age ± SD), with 15 males and 16 females, (b) 32 subjects (18.81 ± .78 years) with 15 males and 17 females, and (c) 33 subjects (18.76 ± .71 years) with 17 males and 16 females.

### MR task

**Figure 3:**
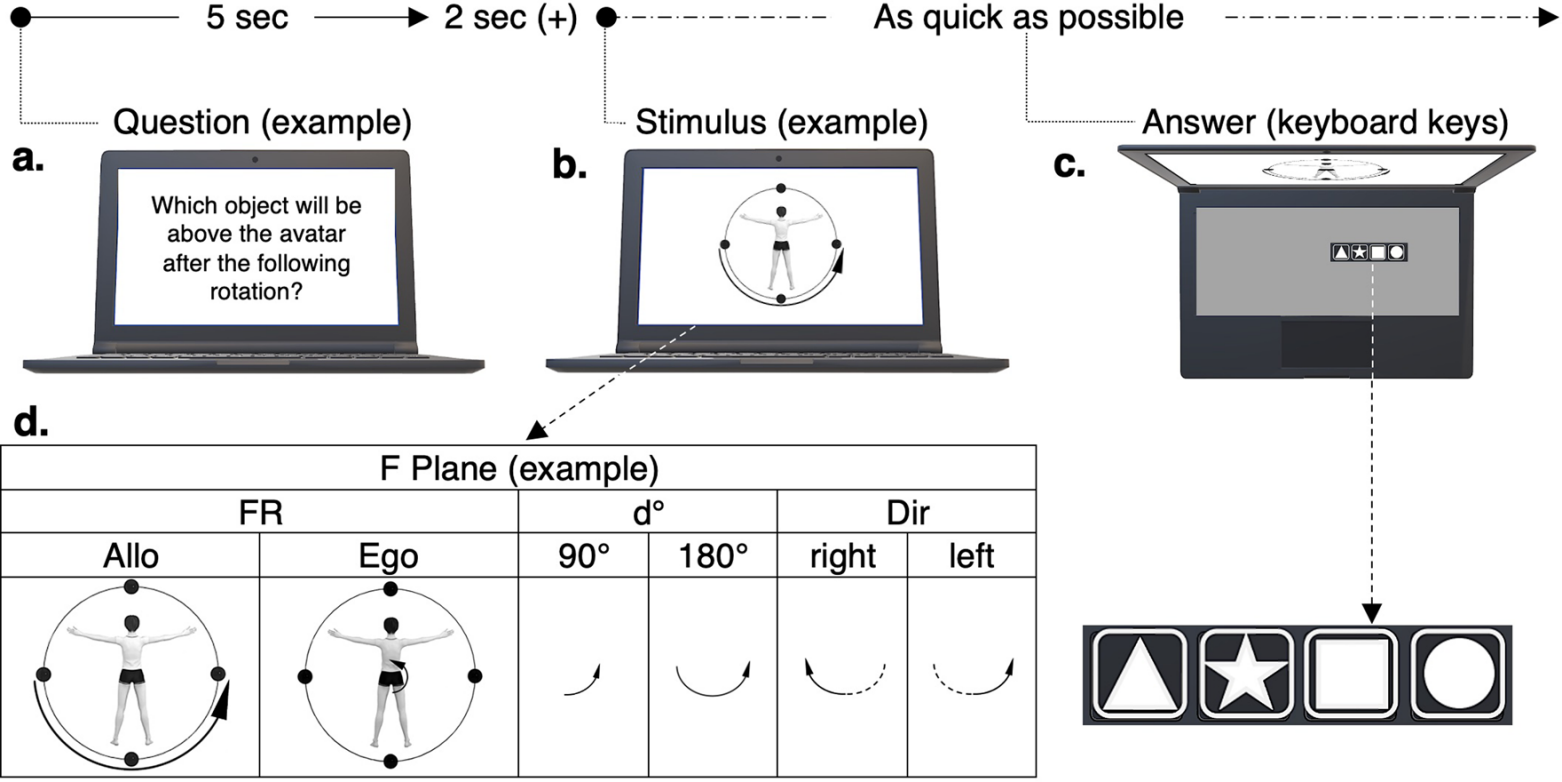
Design of the MRt (MR task) series: Example in F Plane (frontal plane). Each series was performed by using the same laptop, after a phase of familiarization. The subjects performed each MRt by identifying themselves with the avatar shown on the laptop screen, surrounded by a circle on which four black dots were placed to represent the arrangement of four objects that had been memorized during familiarization (a red triangle, a green star, a blue square, and a yellow disc). Before each MRt, a question was shown (for five sec), asking which object would be at a predetermined place relative to the avatar after a given MRt (Fig. 3a). The MRt was then defined by a graphical image (two sec after the question was removed) (Fig. 2b). Once seen the image, the subject answered as quickly as possible by pressing (with the dominant hand) one of four non-hidden keyboard keys that represented the four possible objects (Fig. 3c the objects on the keyboard-keys were the same as on the circle and were put in a clockwise order). The image defined the MRt by giving a set of instructions (Fig. 3d): (**a**) the circle surrounding the avatar indicated the plane of the MR to be done (Plane): either F (as in the example shown in Fig. 3d), the horizontal (H) plane, or the sagittal (S) one, (**b**) the curvilinear arrow placed on the image designated the frame of reference to be used: either allocentric (arrow outside of the circle; Allo), or egocentric (arrow on the avatar; Ego), (**c**) the shape of the arrow curve indicated the degrees of the MR (d°): either 90°, indicated by a quarter-circle, or 180°, indicated by a half-circle and (**d**) the tip of the arrow showed the direction (Dir) of the rotation: either rightward, or leftward in F and H, and either backward, or forward in S. As a consequence, in each Plane hence in each series of the MRt, eight variations of the task were performed (two FR conditions × two d° conditions × two Dir conditions).

The subjects performed three series of MRts (Fig. 3) after a familiarization with a physical device (see: Supplementary Methods/Supplementary Fig. S2 on line). Each MRt was performed in front of the image, shown on the same 13 inches laptop, of an avatar standing upright at the centre of a circle on which four dots were placed in opposition one to another, to represent the arrangement of four different objects (a yellow disk, a red triangle, a blue square, and a green star) that had been memorized during the familiarization procedure (see: Supplementary Methods/Supplementary Fig. S2 on line). In short, the laptop was put on a desk at hip level and the subject stood bent in front of the laptop screen, with the non-dominant hand next to the keyboard. In addition; the subject maintained the fingertips of the dominant hand above four consecutive keyboard keys used to answer.

The plane of the MR (Plane condition), defined by the circle shown on the laptop screen, was varying from one MRt series to another with MR performed in the frontal plane of the avatar (F, shown in Fig. 3 as an example), in the horizontal plane at the feet of the avatar (H) or in the sagittal plane of the avatar (S). The three series were randomly assigned.

In each series the MRts varied randomly (Fig. 3) according to the degrees of the MR (d°: 90°, or 180°), the rotation direction in the plane (Dir: two possible directions in each plane) and the frame of reference used to perform MR (FR: either allocentric, or egocentric). A time lag of three sec separated two consecutive MRts during a series.

The arrangement of the objects at the beginning of the MRt was the same in each series. The four objects on the circle were unambiguously placed, relative to the avatar as: (a) one of the four objects was above the avatar’s head in the F and S plane conditions and in front of the avatar’s toes in the H plane condition, (b) the objects were at the extremities of two orthogonal diameters of the circle, and (c) the MR was either at 90°, or at 180°.

Before each series, the subjects were informed of the task design (Fig. 3) and they were instructed to identify themselves and the device used during the familiarization with the avatar, the circle and the dots on the image shown on the laptop screen. The elements used to define the MRt (Fig. 3) were also explained step-by-step to each subject. On such base, each subject performed five pre-trials. The experiment was beginning only if the subject had performed, at least, three trials correctly. If it was not the case, the task was re-explained, five other pre-trials were performed, and so on.

In each experimental series, i.e.: in each Plane (Fig. 3), a question was written on the screen of the laptop during five sec (the text was centred, written in black on a white background). It was systematically asked which object would be at a given location (as during familiarization; see: Supplementary Methods/Supplementary Fig. S2 on line) and relative to the avatar, after a rotation (see: Fig. 3a). Afterwards, a fixation cross was seen at the centre of the screen for two sec and then the rotation was defined by using an image that showed the avatar, the circle, the four dots placed on the circle and a curvilinear arrow (Fig. 3). In each Plane, this led to define eight versions of the MRt (Fig. 3). Each version was performed twice with initial questions that differed regarding the location of the object to be determined^34^; e.g., in H: (a) in front of the avatar and (b) behind the avatar.

### Measurements

A computer program implemented with Matlab® R2017a (The MathWorks, Inc., Natick, Massachusetts, United States) was created to show the stimuli on the screen and to measure the performances obtained by each subject. This led to collect, in each MRt, the RT from the instant when the MRt was defined on the laptop screen to the instant when a keyboard key was pressed by the subject, whether the answer was correct or not (see: Fig. 3).

The mean RT (± SD) was 2.326 ± 1.142 sec. Specific limits regarding RTs are available in the literature, notably in the case of MR tasks with images of body segments as stimuli^60^ suggesting that the RTs should be comprised within .500 sec and 3.500 sec. In the present study no RT ≤ .500 sec was obtained. However, 14.52% of the RTs were over 3.500 sec and 2.40%, over than 5.000 sec. In the absence of specific limits regarding the RTs in the present study, and considering that the percent of RTs ≥ 5.000 sec was low, it has been decided to include these RTs in the study.

### Statistical analysis

The statistical analysis has been first focused on the RTs of the correct trials as the global error rate (ER) was low (7.55%; for a presentation of the ERs found in the present study, see: Supplementary Tables S15–S20 on line).

A prior log-transformation of the RTs was necessary because their distribution was highly skewed to the right. After the log-transformation the model residuals turned out to be normally distributed (see: Supplementary Fig. S1 on line). The log-transformed RTs (natural logarithms; log RTs) were fitted by a linear mixed-effects model (LMEM)^41^. The model considered the variations in log RT due to the inter-individual variability (Subject effect, as a random effect) and to the intra-individual variability (residual random error). Gender and PA (Sed, Art and Fut) were used as between-subjects fixed effects. Moreover, d° (rotation either at 90° or at 180°), FR (frame of reference to perform the MRt, either Allo or Ego) and PlaneDir (Fr, Fl, Hr, Hl, Sb and Sf) were used as within-subjects fixed effects. In the case of PlaneDir, six different experimental conditions were taken into consideration and not a simple crossing of a plane effect and a direction effect.

The measured RTs were analysed by taking into account these series of variables, which led to an initial model of log RT including all order-one interactions (of fixed-effects). A backward stepwise algorithm of selection then removed non-significant terms at the 5% level (likelihood ratio test) while respecting the “marginality principle”^61^. The quality of the final LMEM was evaluated using the percentage of explained variance, a likelihood ratio test of the Subject random effect, and an analysis of deviance table for the fixed effects. A series of graphical displays called “effect plots”^41^ was used to support the interpretation in the case of interactions. Multiple comparisons were carried out using the methods of Bretz, Hothorn and Westfall^40^; *p*-values were calculated using the single-step method for multiplicity adjustment.

As a complement, the ERs have been taken into consideration by analysing the probabilities of success. A generalized linear mixed-effects model has been used to define the main determinants of the rate of success (all explanatory variables were fixed effects and subject was the only random effects; for more information, see: Supplementary information SI.6. on line).

The statistical analyses were performed using the software R 3.4.3 and the packages nlme, car, effects and multcomp.

## Supplementary Information

Supplementary Information.

## Data Availability

The data analysed in the present study are available from the corresponding author.
