# Supplementary material for: Modelling response time in a mental rotation task by gender, physical activity, and task features
Source: Sci Rep. 2022 Sep 16;12:15559. doi: 10.1038/s41598-022-19054-2 (PMC9481519; doi:10.1038/s41598-022-19054-2)
Supplement: Supplementary file 1 — Supplementary Information. [file 41598_2022_19054_MOESM1_ESM.pdf]

Modelling response time in a mental rotation task by gender, physical activity, and task features

Patrick Fargier[1,2,*], Stéphane Champely[3,+], Raphael Massarelli[4,+], Laureine Ammary[2], and Nady Hoyek[2]

[1]University of Teacher Education, State of Vaud (HEPVD), UER EPS, Lausanne, 1014, Switzerland.
[2]Univ Lyon, University Claude Bernard Lyon 1, Inter-university Laboratory on Human Movement Biology (LIBM), Villeurbanne, 69622, France.
[3] Univ Lyon, University Claude Bernard Lyon 1, Laboratory of Vulnerabilities and Innovation in Sport (L-Vis), Villeurbanne, 69622, France.
[4]Univ Lyon, University Claude Bernard Lyon 1 (work carried out as Honorary Professor), Villeurbanne, 69622, France.
*corresponding author: patrick.fargier@hepl.ch
+these authors contributed equally to this work

Supplementary information (SI)

SI.1. Response times (RTs)

The RTs (sec) obtained by the subjects are shown below, considering:

- The between-subjects variables: 1. Gender [female (Fe) and male (Ma) subjects], and 2. PA [physical activity regularly practiced by the subjects who were sedentary subjects (Sed), artistic gymnasts (Art), or futsal players (Fut)];
- The within-subjects variables: 1. d° (degrees of the rotation to be imagined, either 90°, or 180°), 2. PlaneDir [plane and direction of the rotation to be imagined, rotation in the frontal plane in the right direction (Fr) or in the left direction (Fl), rotation in the horizontal plane in the right direction (Hr) or in the left direction (Hl), or rotation in the sagittal plane backward (Sb) or forward (Sf) directed, and 3. FR [frame of reference used to imagine the rotation, allocentric FR (Allo) or egocentric FR (Ego)];
- The variables involved in each interaction effect found in the study: 1. PA*d°, 2. PlaneDir*PA, 3. PlaneDir*d°, and 4. PlaneDir*FR.

SI.1.1. RTs when considering the between-subjects variables

	**Gender**		**PA**		
	**Fe**	**Ma**	**Sed**	**Art**	**Fut**
**Meam RT**	2.53	2.11	2.78	2.23	1.99
**SD**	1.22	1.01	1.37	.87	.97

Table S1. Mean RTs obtained when considering the between-subjects variables of the study.

SI.1.2. RTs when considering the within-subjects variables

	d°		PlaneDir						FR	
	90°	180°	Fr	Fl	Hr	Hl	Sb	Sf	Allo	Ego
**Mean RT**	2.50	2.16	2.60	2.43	1.97	1.93	2.46	2.56	2.30	2.35
**SD**	1.05	1.20	1.30	1.13	.92	.90	1.10	1.24	1.11	1.17

Table S2. Mean RTs obtained when considering the within-subjects variables of the study.

*SI.1.3. RTs when considering the variables involved in PA*d°*

PA	Sed		Art		Fut	
**d°**	**90°**	**180°**	**90°**	**180°**	**90°**	**180°**
**Mean RT**	2.75	2.81	2.33	2.12	2.38	1.59
**SD**	1.23	1.50	.87	2.40	.97	.81

Table S3. Mean RTs obtained when crossing the variables involved in d°*PA.

*SI.1.4 RTs when considering the variables involved in PlaneDir*PA, PlaneDir*d°, and PlaneDir*FR*

PA	Sed					
**PlaneDir**	**Fr**	**Fl**	**Hr**	**Hl**	**Sb**	**Sf**
**Mean RT**	3.28	2.82	2.21	2.18	2.95	3.28
**SD**	1.51	1.28	1.09	1.02	1.29	1.54
**PA**	**Art**					
**PlaneDir**	**Fr**	**Fl**	**Hr**	**Hl**	**Sb**	**Sf**
**Mean RT**	2.39	2.32	2.07	1.98	2.23	2.36
**SD**	.87	.86	.86	.84	.88	.87
**PA**	**Fut**					
**PlaneDir**	**Fr**	**Fl**	**Hr**	**Hl**	**Sb**	**Sf**
**Mean RT**	2.15	2.15	1.65	1.64	2.23	2.08
**SD**	1.17	1.13	.71	.76	.93	.91

Table S4. Mean RTs obtained when crossing the variables involved in PlaneDir*PA.

d°	90°					
**PlaneDir**	**Fr**	**Fl**	**Hr**	**Hl**	**Sb**	**Sf**
**Mean RT**	2.90	2.76	2.15	2.09	2.49	2.53
**SD**	1.14	1.09	.93	.89	1.00	.99
**d°**	**180°**					
**PlaneDir**	**Fr**	**Fl**	**Hr**	**Hl**	**Sb**	**Sf**
**Mean RT**	2.31	2.09	1.79	1.77	2.43	2.59
**SD**	1.37	1.08	.88	.89	1.18	1.46

Table S5. Mean RTs obtained when crossing the variables involved in PlaneDir*d°.

FR	Allo					
**PlaneDir**	**Fr**	**Fl**	**Hr**	**Hl**	**Sb**	**Sf**
**Mean RT**	2.56	2.29	2.03	2.00	2.47	2.48
**SD**	1.20	1.16	.96	.96	1.07	1.17
**FR**	**Ego**					
**PlaneDir**	**Fr**	**Fl**	**Hr**	**Hl**	**Sb**	**Sf**
**Mean RT**	2.62	2.56	1.91	1.87	2.45	2.64
**SD**	1.39	1.09	.88	.85	1.12	1.31

Table S6. Mean RTs obtained when crossing the variables involved in PlaneDir*FR.

SI.2. Normal quantile plot of the residuals after the log-transformation of the RTs

In the present study, the distribution of the RTs was highly skewed to the right. A log-transformation of the RTs was done in order to develop the statistical model. After the log-transformation, the model residuals were found to be normally distributed (see: Supplementary Figure S7).

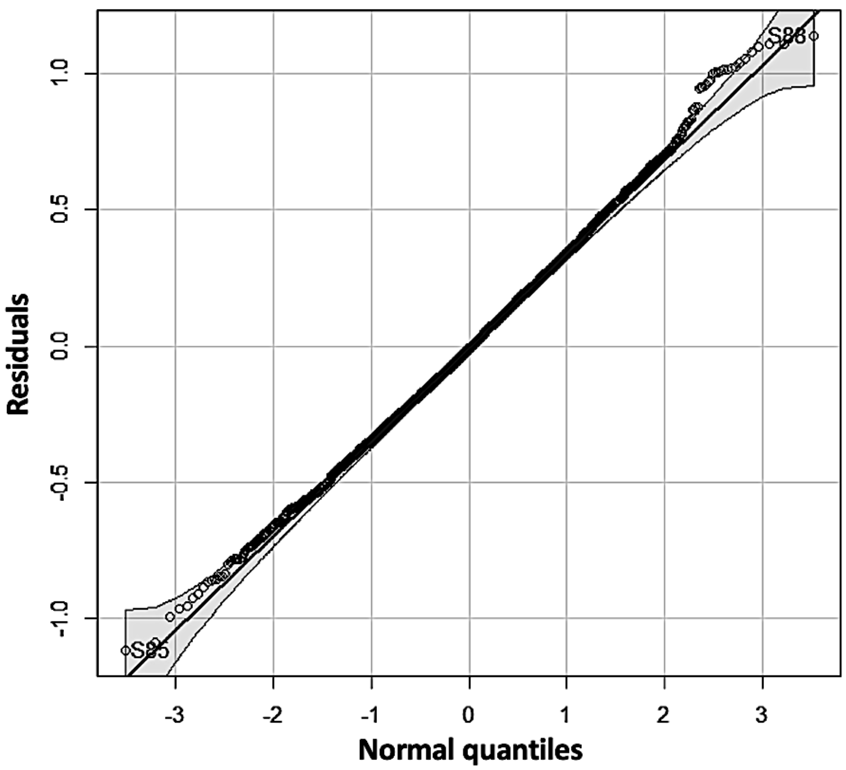

Figure S1. Normal quantile plot of the residual after the log-transformation (natural logarithm) of the RTs.

SI.3. Comparisons of log RTs

The selected model of the log response-times (log RTs) obtained by the subjects included four interaction (*) effects that were statistically significant:

(1) PA*d° (interaction between the physical activity practiced by the subjects and the degrees of the rotation to be imagined);
(2) PlaneDir*PA (interaction between the plane and direction of the rotation to be imagined and PA);
(3) PlaneDir*d°;
(4) PlaneDir*FR (interaction between PlaneDir and the frame of reference used to imagine the rotation).

The examination of each interaction led to a series of comparisons of the log RTs that are detailed below. The multiple comparisons were carried out using the method of Bretz, Hothorn, and Westfall[1] (family-wise error rate set at 5%). Regarding the Estimates (differences in log RT) the values of two standard errors may differ after the third decimal only. The corresponding z-scores and adjusted p-values [single-step method; p (>|z|)] are also given.

It should be noted that the difference between two log RTs (Estimate) gives the percentage of variation (PV) from one RT to the other with PV = 100 × [exp(Estimate) - 1].

*SI.3.1. PA*d°*

Difference	Estimate (log RT)	Standard error	*z*-score	*p* (>|*z*|)
**Sed**				
**90° - 180°**	.01	.03	.37	.975
**Art**				
**90° - 180°**	.11	.05	4.38	3.53e-5
**Fut**				
**90° - 180°**	.43	.02	16.92	< 1e-15

Table S7. Comparisons of log RTs between the two d° conditions (degrees of the rotation to be imagined, *i.e.*: either 90°, or 180°) of the MR task, according to PA (physical activity: Sed, sedentary subjects; Art, artistic gymnasts and Fut, futsal players).

Difference	Estimate (log RT)	Standard error	*z*-score	*p* (>|*z*|)
**90°**				
**Sed - Fut**	.12	.05	2.226	.107
**Sed - Art**	.14	.05	2.580	.044
**Fut - Art**	.02	.05	.371	.988
**180°**				
**Sed - Fut**	.54	.05	10.02	< 1e-15
**Sed - Art**	.24	.05	4.47	5.73e-5
**Fut - Art**	-.30	.05	-5.57	7.21e-8

Table S8. Comparisons of log RTs among the PA groups, according to the d° conditions of the MR task. Results of the multiple comparisons of log RTs among the PA (physical activity) groups (Sed, sedentary subjects; Fut, futsal players and Art, artistic gymnasts) and according to each d° condition (degrees of the imagined rotation: either 90° or 180°).

*SI.3.2. PlaneDir*PA*

Difference	Estimate (log RT)	Standard error	*z*-score	*p* (>|*z*|)
**Sed**				
**F - H**	.33	.03	10.25	< 1e-15
**F - S**	-.03	.03	-.98	.998
**H - S**	-.36	.03	-11.20	< 1e-15
**Fr - Fl**	.16	.04	3.62	.005
**Hr - Hl**	.01	.04	.29	1.000
**Sb - Sf**	-.10	.04	-2.20	.378
**Art**				
**F - H**	.17	.03	5.28	2.40e-6
**F - S**	.03	.03	.98	.998
**H - S**	-.13	.03	-4.29	3.15e-4
**Fr - Fl**	.03	.04	.76	.100
**Hr - Hl**	.05	.04	1.08	.995
**Sb - Sf**	-.06	.04	-1.42	.927
**Fut**				
**F - H**	.23	.03	7.38	2.87e-12
**F - S**	-.04	.03	-1.24	.979
**H - S**	-.27	.03	-8.69	< 1e-15
**Fr - Fl**	.002	.04	.05	1.000
**Hr - Hl**	.01	.04	.34	1.000
**Sb - Sf**	.07	.04	1.69	.789

Table S9. Comparisons of MR task performances (log RTs) among Plane conditions and between the two Dirs in each Plane condition, according to PA. The Plane conditions are the planes in which the rotation was imagined: frontal (F), horizontal (H) and sagittal (S) planes. The Dirs are the rotation directions in F: right (Fr) or left (Fl), in H: Hr or Hl, and in S: backward (Sb) or forward (Sf). PA is the physical activity practised by the subjects (sedentary subjects, Sed; artistic gymnasts, Art and futsal players, Fut).

Difference	Estimate (log RT)	Standard error	*z*-score	*p* (>|*z*|)
**F**				
**Sed - Fut**	.37	.06	6.42	2.11e-9
**Sed - Art**	.22	.06	3.92	.001
**Fut - Art**	-.14	.06	-2.49	.168
**H**				
**Sed - Fut**	.27	.06	4.74	3.82e-5
**Sed - Art**	.06	.06	1.11	.974
**Fut - Art**	-.21	.06	-3.65	.004
**S**				
**Sed - Fut**	.36	.06	6.30	4.03e-9
**Sed - Art**	.29	.06	5.00	8.46e-6
**Fut - Art**	-.07	.06	-1.27	.935
**Fr - Fl**				
**Sed - Fut**	.16	.06	2.55	.148
**Sed - Art**	.13	.06	2.05	.427
**Fut -Art**	-.03	.06	-.50	1.000
**Hr - Hl**				
**Sed - Fut**	-.002	.06	-.04	1.000
**Sed - Art**	-.03	.06	-.55	1.000
**Fut - Art**	-.03	.06	-.52	1.000
**Sb - Sf**				
**Sed - Fut**	-.17	.06	-2.76	.084
**Sed - Art**	-.04	.06	-0.56	1.000
**Fut - Art**	.14	.06	2.21	.314

Table S10. Comparisons of MR task performances (log RTs) among PA groups, according to the Plane conditions and the Dirs in each Plane condition. The PA (physical activity) groups are: Sed (sedentary subjects), Fut (futsal players) and Art (artistic gymnasts). The Plane conditions are the planes in which the rotation was imagined: frontal (F), horizontal (H) and sagittal (S) planes. The Dir conditions are the rotation directions in F: right (Fr) or left (Fl); in H: Hr or Hl and in S: backward (Sb) or forward (Sf).

*SI.3.3. PlaneDir*d°*

Difference	Estimate (log RT)	Standard error	*z*-score	*p* (>|*z*|)
**90°**				
**F - H**	.30	.03	11.55	< 1e-15
**F - S**	.12	.03	4.60	5e-5
**H - S**	-.18	.03	-6.99	2.91e-11
**Fr - Fl**	.055	.04	1.49	.790
**Hr - Hl**	.032	.04	.88	.993
**Sb - Sf**	-.018	.04	-.50	1.000
**180°**				
**F - H**	.18	.03	7.11	1.35e-11
**F - S**	-.14	.03	-5.60	2.55e-7
**H - S**	-.33	.03	-12.70	< 1e-15
**Fr - Fl**	.07	.04	2.07	.352
**Hr - Hl**	.02	.04	.52	1.000
**Sb - Sf**	-.04	.04	-1.06	.972

Table S11. Comparisons of MR task performances (log RTs) among Plane conditions and between Dirs in each Plane condition, according to d°. The Plane conditions are the planes in which the rotation was imagined: frontal (F), horizontal (H) and sagittal (S). The Dirs are the rotation directions in F: right (Fr) or left (Fl); in H: Hr or Hl and in S: backward (Sb) or forward (Sf). d° denotes the degrees of the imagined rotation: either 90° or 180°.

Difference	Estimate (log RT)	Standard error	*z*-score	*p* (>|*z*|)
**F**				
**90° - 180°**	.31	.03	12.18	< 1e-15
**H**				
**90° - 180°**	.19	.03	7.71	7.44e-14
**S**				
**90° - 180°**	.05	.03	2.01	.237
**Fr – Fl**				
**90° - 180°**	-.02	.05	-.39	.999
**Hr - Hl**				
**90° - 180°**	.01	.05	.26	1.000
**Sb - Sf**				
**90° - 180°**	.02	.05	.40	.999

Table S12. Comparison of MR task performances (log RTs) between d° conditions, according to Plane and Dir. d° denotes the degrees of the imagined rotation: 90° or 180°. The Plane conditions are the planes of the imagined rotations: frontal (F), horizontal (H) and sagittal (S). The Dirs are the rotation directions in F: right (Fr) or left (Fl); in H: Hr or Hl and in S: backward (Sb) or forward (Sf).

*SI.3.4. PlaneDir*FR*

**Difference**	**Estimate (log RT)**	**Standard error**	***z*-score**	***p* (>|*z*|)**
**Allo**				
**F - H**	.17	.03	6.78	1.36e-10
**F - S**	-.03	.03	-1.39	.867
**H - S**	-.21	.03	-8.17	4.88e-15
**Fr - Fl**	.12	.04	3.26	.013
**Hr - Hl**	.03	.04	.89	.993
**Sb - Sf**	.015	.04	.41	1.000
**Ego**				
**F - H**	.31	.03	11.88	< 1e-15
**F - S**	.01	.03	.36	1.000
**H - S**	-.30	.03	-11.53	< 1e-15
**Fr - Fl**	.01	.04	.30	1.000
**Hr - Hl**	.02	.04	.51	1.000
**Sb - Sf**	-.07	.04	-1.98	.419

Table S13. Comparison of MR task performances (log RTs) among Plane conditions and between the two Dirs in each Plane, according to FR. The Plane conditions are the planes of the imagined rotations, *i.e.*: frontal (F), horizontal (H) or sagittal (S) plane. The Dirs are on the right or on the left direction in F (respectively: Fr and Fl) and in H (respectively: Hr and Hl) and backward or forward in S (respectively: Sb and Sf). FR is the frame of reference to imagine rotation: allocentric (Allo) or Egocentric (Ego).

**Difference**	**Estimate (log RT)**	**Standard error**	***z*-score**	***p* (>|*z*|)**
**F**				
**Allo - Ego**	-.08	.03	-3.10	.012
**H**				
**Allo - Ego**	.05	.03	2.00	.245
**S**				
**Allo - Ego**	-.03	.03	-1.38	.668
**H – F**				
**Allo - Ego**	.13	.04	3.60	.002
**S – F**				
**Allo - Ego**	.04	.04	1.22	.736
**S – H**				
**Allo - Ego**	-.09	.04	-2.39	.092
**Fr**				
**Allo - Ego**	-.03	.04	-.70	.980
**Fl**				
**Allo - Ego**	-.13	.04	-3.68	.001
**Hr**				
**Allo - Ego**	.06	.04	1.60	.501
**Hl**				
**Allo - Ego**	.04	.04	1.22	.776
**Sb**				
**Allo - Ego**	.01	.04	.22	1.00
**Sf**				
**Allo - Ego**	-.08	.04	-2.17	.167
**Fr - Fl**				
**Allo - Ego**	.11	.05	2.10	.196
**Hr - Hl**				
**Allo - Ego**	.01	.05	.27	1.000
**Sb - Sf**				
**Allo - Ego**	.09	.05	1.70	.435

Table S14. Comparisons of MR task performances (log RTs) between FR conditions (frame of reference to imagine rotation, *i.e.*: allocentric, Allo and egocentric, Ego). The differences (Allo - Ego) in log RTs according to each Plane condition [plane of imagined rotation: frontal (F), horizontal (H) or sagittal (S)] and the variations, among the Plane conditions, of the differences are shown. The differences (Allo - Ego) in log RTs according to each Dir in each Plane condition [direction of the rotation in each Plane condition: in F, right (Fr) or left (Fl) direction, in H, right (Hr) or left (Hl) direction and in S, forward (Sf) or backward (Sb) direction] and the variations of the differences between the two Dirs in each Plane condition are also shown.

SI.4 – Calculation of the ΔMs

The ΔMs concerned the differences among the mean log RTs obtained in the horizontal, frontal, and sagittal planes. The mean log RTs were calculated considering:

- In the horizontal plane: the log RTs obtained when the mental rotation was performed in the left direction and when it was performed in the right direction;
- In the frontal plane: the log RTs obtained when the mental rotation was performed in the left direction and when it was performed in the right direction;
- In the sagittal plane: the log RTs obtained when the mental rotation performed was backward directed and when it was forward directed.

Using the estimation of the parameters, the means were calculated just as in an effect plot[2]. The differences were obtained as a linear function of these means and simultaneous inference was carried out using the compmult package based on Bretz, Hothorn, and Westfall[1].

SI.5. Error rates (ERs)

The ERs (as percent) of the subjects are shown below, considering:

- The between-subjects variables: 1. Gender [female (Fe) and male (Ma) subjects], and 2. PA [physical activity regularly practised by the subjects who were sedentary subjects (Sed), artistic gymnasts (Art), or futsal players (Fut)];
- The within-subjects variables: 1. d° (degrees of the rotation to be imagined, either 90°, or 180°), 2. PlaneDir [plane and direction of the rotation to be imagined, rotation in the frontal plane in the right direction (Fr) or in the left direction (Fl), rotation in the horizontal plane in the right direction (Hr) or in the left direction (Hl), or rotation in the sagittal plane backward (Sb) or forward (Sf) directed, and 3. FR [frame of reference used to imagine the rotation, allocentric FR (Allo) or egocentric FR (Ego)];
- The variables involved in each interaction effect found in the study: 1. PA*d°, 2. PlaneDir*PA, 3. PlaneDir*d°, and 4. PlaneDir*FR.

SI.5.1. ERs when considering the between-subjects variables

	**Gender**		**PA**		
	**Fe**	**Ma**	**Sed**	**Art**	**Fut**
**ER (%)**	8.15	5.83	10.96	2.99	7.20

Table S15. Error rates when considering the between-subjects variables of the study.

SI.5.2. ERs when considering the within-subjects variables

	**d°**		**PlaneDir**						**FR**	
	**90°**	**180°**	**Fr**	**Fl**	**Hr**	**Hl**	**Sb**	**Sf**	**Allo**	**Ego**
**ER (%)**	5.95	8.06	8.44	8.05	3.22	2.44	9.40	10.53	7.97	6.05

Table S16. Error rates when considering the within-subjects variables of the study.

*SI.5.3. ERs when considering the variables involved in PA*d°*

**PA**	**Sed**		**Art**		**Fut**	
**d°**	**90°**	**180°**	**90°**	**180°**	**90°**	**180°**
**ER (%)**	6.72	15.22	2.60	3.38	8.54	5.89

Table S17. Error rates when crossing the variables involved in d°*PA.

*SI.5.4. ERs when considering the variables involved in PlaneDir*PA, PlaneDir*d°, and PlaneDir*FR*

**PA**	**Sed**					
**PlaneDir**	**Fr**	**Fl**	**Hr**	**Hl**	**Sb**	**Sf**
**ER (%)**	12.90	13.44	4.30	3.22	15.18	16.80
**PA**	**Art**					
**PlaneDir**	**Fr**	**Fl**	**Hr**	**Hl**	**Sb**	**Sf**
**ER (%)**	3.38	2.86	2.34	1.82	2.86	4.69
**PA**	**Fut**					
**PlaneDir**	**Fr**	**Fl**	**Hr**	**Hl**	**Sb**	**Sf**
**ER (%)**	9.19	8.01	3.05	2.29	10.35	10.35

Table S18. Error rates when crossing the variables involved in PlaneDir*PA.

**d°**	**90°**					
**PlaneDir**	**Fr**	**Fl**	**Hr**	**Hl**	**Sb**	**Sf**
**ER (%)**	6.59	6.70	3.49	2.09	8.51	8.33
**d°**	**180°**					
**PlaneDir**	**Fr**	**Fl**	**Hr**	**Hl**	**Sb**	**Sf**
**ER (%)**	10.24	9.37	2.95	2.78	10.30	12.74

Table S19. Error rates when crossing the variables involved in PlaneDir*d°.

**FR**	**Allo**					
**PlaneDir**	**Fr**	**Fl**	**Hr**	**Hl**	**Sb**	**Sf**
**ER (%)**	8.99	9.82	3.82	2.95	10.07	12.22
**FR**	**Ego**					
**PlaneDir**	**Fr**	**Fl**	**Hr**	**Hl**	**Sb**	**Sf**
**ER (%)**	7.89	6.28	2.62	1.92	8.72	8.85

Table S20. Error rates when crossing the variables involved in PlaneDir*FR.

SI.6. Analysis of the probabilities of success

The analysis focused on the probability of success to approach the error rates. A generalized linear mixed effects model[3] was employed to define the main determinants of the rate of success.
The outcome binary variable (success vs failure) was fitted using the binomial family and the logit link function. All explanatory variables were fixed effects and subject was the only random effects. The variables were:

- Between-subjects variables: 1. Gender [female (Fe) and male (Ma) subjects], and 2. PA [physical activity regularly practiced by the subjects who were sedentary subjects (Sed), artistic gymnasts (Art), or futsal players (Fut)];
- Within-subjects variables: 1. d° (degrees of the rotation to be imagined, either 90°, or 180°), 2. PlaneDir [plane and direction of the rotation to be imagined, rotation in the frontal plane in the right direction (Fr) or in the left direction (Fl), rotation in the horizontal plane in the right direction (Hr) or in the left direction (Hl), or rotation in the sagittal plane backward (Sb) or forward (Sf) directed, and 3. FR [frame of reference used to imagine the rotation, allocentric FR (Allo) or egocentric FR (Ego)].

Additive effects and order-one interactions were considered for fixed effects.

The statistically significant main effects and interaction effects, when ranked in decreasing order of importance using the Chi^{sq}/df criterion of McCullagh and Nelder[4] were the following: PA*d° (Chi^{sq}/df = 19.7; $p < .001$), PlaneDir (Chi^{sq}/df = 18.8; $p < .001$), FR*d° (Chi^{sq}/df = 11.8; $p < .001$), Gender (Chi^{sq}/df = 11.2; $p < .001$). The effect-plots for PA*d°, PlaneDir, FR*d°, and Gender are shown by the Figure S3.

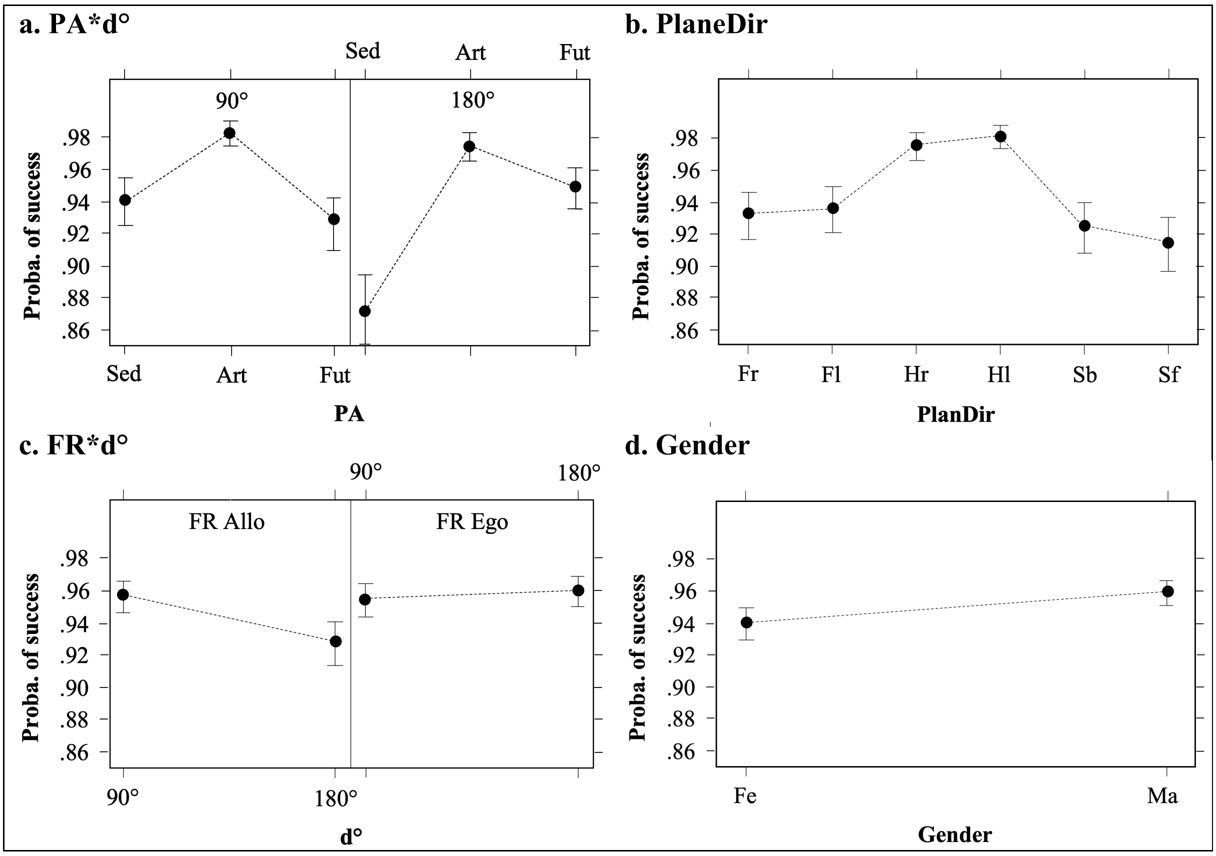

Figure S2. Interaction effects and main effects among Gender, PA, Degree, PlaneDir, and FR on the probability of success (Proba.). The effect plots[5] for PA*d° (a.), PlaneDir (b.), FR*d° (c.), and Gender (d.) are ranked in decreasing order of importance according to the Chi^{sq}/df criterion[4]. The effect-plot a. shows the Proba.s in each PA (physical activity) group (sedentary subjects, Sed, artistic gymnasts, Art, and futsal players, Fut) according to the degrees of the rotation imagined (d°: 90° or 180°). The effect-plot b. shows Proba.s according to each PlaneDir condition (plane and direction in which the rotation was imagined), *i.e.*: rotation in the frontal plane on the right (Fr) or on the left (Fl), in the horizontal plane on the right (Hr) or on the left (Hl), in the sagittal plane on a backward direction (Sb) or forward (Sf). The effect-plot c. shows the Proba.s in each FR condition (frame of reference to imagine rotation), *i.e.*: either allocentric (Allo) or egocentric (Ego), according to d°. The effect-plot d. shows the Proba.s according to Gender, *i.e.*: Fe (female subjects) and Ma (male subjects). In each effect-plot, the vertical line segments indicate ±95% confidence interval and broken lines connect the Proba.s values to favour visual comparisons and to emphasize the interactions for PA*d° and FR*d°.

Estimated marginal means (Emmeans) were computed in order to carry out post-hoc test for the different main effects and interaction effects using Tukey's all pairwise comparisons.
In the following, the Emmeans results are given on the logit (not the response) scale; the confidence level used was .95. Considering the value Mn for a given Emmean, the corresponding probability of success (Proba.) may be computed as follows: Proba. = 1 / [1+exp(-Mn)], as Emmean = log[Proba./(1-Proba.)].
In addition, the obtained comparisons are given as log odds ratios (differences between logit values).

*SI.6.1. PA*d°*

Table S21 shows the estimated marginal means concerning PA*d°.

**d°**	**90°**			**180°**		
**PA**	**Emmean**	**SE**	**Proba.**	**Emmean**	**SE**	**Proba.**
**Sed**	2.84	.140	.94	1.92	.111	.87
**Art**	3.87	.201	.98	3.64	.182	.97
**Fut**	2.56	.127	.93	3.02	.145	.95

Table S21. Probabilities of success (Proba.s) for PA according to d°. The Proba.s are given for each PA (physical activity) group (sedentary subjects, Sed, artistic gymnasts, Art, and futsal players, Fut) according to the degrees of the rotation imagined (d°: 90° or 180°). Results are averaged over the levels of: Gender, FR, and PlaneDir. Each Proba. has been computed from the corresponding estimated marginal mean (Emmean); the values of the Emmeans (± standard error, SE) are given.

Table S22 shows the comparisons of the Emmeans between the two d° conditions according to the PA groups.

**Contrasts**	**Estimate**	**SE**	**z-ratio**	***p***
**Sed**				
**90° - 180°**	.925	.149	6.213	< .0001
**Art**				
**90° - 180°**	.231	.248	.931	.3519
**Fut**				
**90° - 180°**	-.464	.165	-2.817	.0049

Table S22. Comparisons (Estimates) of the Estimated marginal means between the two d° conditions (90° or 180°) according to Sed (sedentary subjects), Art (artistic gymnasts), and Fut (futsal players). Results are averaged over the levels of: Gender, FR, and PlaneDir. Results are given on the log odds ratio (not the response) scale. The Estimates (± standard error, SE), the z-ratios, and the *p*-values are given.

Table S23 shows the comparisons of the Emmeans among the PA (physical activity) groups according to d° (degrees of the rotation imagined).

**d°**	**90°**				**180°**			
**Contrasts**	**Estimate**	**SE**	**z-ratio**	***p***	**Estimate**	**SE**	**z-ratio**	***p***
**Art - Sed**	1.031	.239	4.311	< .0001	1.725	.207	8.349	< .0001
**Fut - Sed**	-.285	.184	-1.555	.2656	1.104	.176	6.281	< .0001
**Art - Fut**	1.317	.232	5.676	< .0001	.622	.225	2.769	.0155

Table S23. Comparisons (Estimates) of the Estimated marginal means among Sed (sedentary subjects), Art (artistic gymnasts), and Fut (futsal players) according to the degrees of the rotation imagined (d°: 90° or 180°). Results are averaged over the levels of: Gender, FR, and PlaneDir. Results are given on the log odds ratio (not the response) scale. The Estimates (± standard error, SE), the z-ratios, and the *p*-values are given. Tukey method for comparing a family of three Estimates was used for *p*-values adjustments.

SI6.2. PlaneDir

Table S24 shows the estimated marginal means concerning PlaneDir.

PlaneDir	Emmean	SE	Proba.
**Fr**	2.63	.120	.93
**Fl**	2.68	.122	.94
**Hr**	3.69	.177	.98
**Hl**	3.98	.200	.98
**Sb**	2.51	.115	.92
**Sf**	2.37	.110	.91

Table S24. Probabilities of success (Proba.s) for each PlaneDir condition. The PlaneDir conditions led to rotation: in the frontal plane on the left (Fl) or on the right (Fr), in the horizontal plane on the left (Hl) or on the right (Hr), in the sagittal plane on a backward direction (Sb) or forward (Sf). Results are averaged over the levels of: PA, d°, FR, and Gender.

Table S25 shows the comparisons of the Emmeans between each pair of PlaneDir conditions.

Contrasts	Estimate	SE	z-ratio	*p*
**Fl - Fr**	.057	.156	.365	.999
**Fl - Hl**	-1.302	.222	-5,854	< .0001
**Fl - Hr**	-1.010	.202	-5.005	< .0001
**Fl - Sb**	.175	.152	1.151	.860
**Fl - Sf**	.313	.149	2.103	.285
**Fr - Hl**	-1.359	.221	-6.146	< .0001
**Fr - Hr**	-1.067	.201	-5.314	< .0001
**Fr - Sb**	.118	.151	.785	.970
**Fr - Sf**	.256	.147	1.738	.507
**Hl - Hr**	.292	.255	1.145	.863
**Hl - Sb**	1.477	.218	6.762	< .0001
**Hl - Sf**	1.615	.216	7.468	< .0001
**Hr - Sb**	1.185	.198	5.990	< .0001
**Hr - Sf**	1.322	.195	6.771	< .0001
**Sb - Sf**	.138	.143	.962	.930

Table S25. Comparisons (Estimates) of the Estimated marginal means among the PlaneDir conditions. These conditions led to rotation: in the frontal plane on the left (Fl) or on the right (Fr), in the horizontal plane on the left (Hl) or on the right (Hr), in the sagittal plane on a backward (Sb) or forward direction (Sf). Results are given on the log odds ratio (not the response) scale. The Estimates (± standard error, SE), the z-ratios, and the *p*-values are given. Tukey method for comparing a family of six Estimates was used for *p*-values adjustments.

*SI.6.3. FR*d°*

Table S26 shows the estimated marginal means concerning FR*d°.

FR	Allo			Ego		
**d°**	**Emmean**	**SE**	**Proba.**	**Emmean**	**SE**	**Proba.**
**90°**	3.12	.123	.96	3.06	.120	.96
**180°**	2.55	.103	.93	3.17	.123	.96

Table S26. Probabilities of success (Proba.s) for d° (90°, or 180°) according to FR [allocentric (Allo), or egocentric (Ego)]. Results are averaged over the levels of: PA, PlaneDir, and Gender. Each Proba. has been computed from the corresponding estimated marginal mean (Emmean); the values of the Emmeans (± standard error, SE) are given.

Table S27 shows the comparisons of the Emmeans between the two d° conditions (degrees of the rotation imagined) according to the two FR conditions (frame of reference to imagine rotation).

Contrasts	Estimate	SE	z-ratio	*p*
**Allo**				
**90° - 180°**	.571	.143	3.989	.0001
**Ego**				
**90° - 180°**	-.110	.155	-.707	.478

Table S27. Comparisons (Estimates) of the Estimated marginal means between the two d° conditions (90°, or 180°) according to the two FR conditions [allocentric (Allo), or egocentric (Ego)]. Results are averaged over the levels of: PA, Gender, and PlaneDir. Results are given on the log odds ratio (not the response) scale. The Estimates (± standard error, SE), the z-ratios, and the *p*-values are given.

Table S28 shows the comparisons of the Emmeans between the two FR conditions (frame of reference to imagine rotation) according to the two d° conditions (90°, or 180°).

**Contrasts**	**Estimate**	**SE**	**z-ratio**	***p***
**90°**				
**Allo - Ego**	.061	.147	.414	.679
**180°**				
**Allo - Ego**	-.620	.133	-4.646	< .0001

Table S28. Comparisons (Estimates) of the Estimated marginal means between the two FR conditions [allocentric (Allo), or egocentric (Ego)] according to the two d° conditions (90°, or 180°). Results are averaged over the levels of: PA, Gender, and PlaneDir. Results are given on the log odds ratio (not the response) scale. The Estimates (± standard error, SE), the z-ratios, and the *p*-values are given.

SI.6.4. Gender

Table S29 shows the estimated marginal means concerning Gender.

**Gender**					
**Fe**			**Ma**		
**Emmean**	**SE**	**Proba.**	**Emmean**	**SE**	**Proba.**
2.77	.092	.94	3.19	.104	.96

Table S29. Probabilities (Proba.) of success for Gender [gender of the subjects, either female subjects (Fe), or male subjects (Ma)]. Results are averaged over the levels of: PA, d°, FR, and PlaneDir. Each Proba. has been computed from the corresponding estimated marginal mean (Emmean); the values of the Emmeans (± standard error, SE) are given.

Table S30 shows the comparisons of the Emmeans between the two Gender groups [female subjects (Fe), and male subjects (Ma)].

**Contrasts**	**Estimate**	**SE**	**z-ratio**	***p***
**Fe - Ma**	-.418	.125	-3.344	.0008

Table S30. Comparisons (Estimates) of the Estimated marginal means between the two Gender groups [female subjects (Fe), and male subjects (Ma)]. Results are averaged over the levels of: PA, d°, FR, and PlaneDir. Results are given on the log odds ratio (not the response) scale. The Estimates (± standard error, SE), the z-ratios, and the *p*-values are given.

SI.7 – Supplementary Methods: Familiarization with the experimental MR task

Before each of the three experimental series that have been performed in the present study, the subjects were individually familiarized with the series (Figure S2) after they were informed that this familiarization was prefiguring the performance of a MRt series.

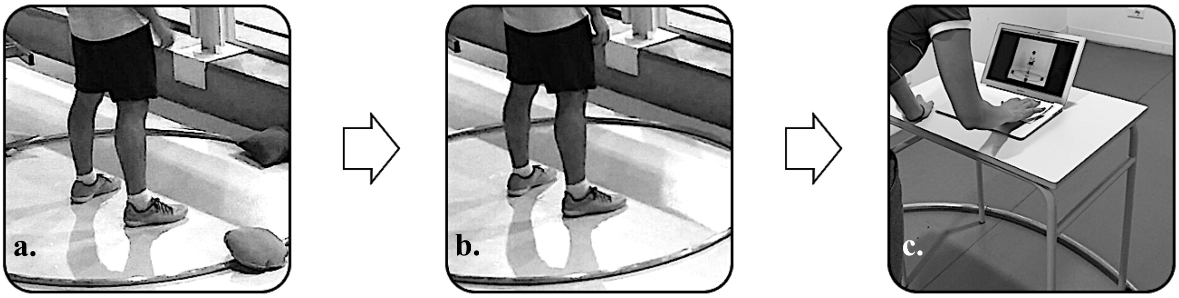

Figure S3. Familiarization with the experimental MR task (MRt). Three sequences, each including a familiarization phase (a. and b.) directly followed by a MRt series (c.), have been performed. From one sequence to another, both the familiarization and the consecutive MRt series varied according to the plane of the rotation that was (relative to the subject and, thereafter, to the avatar): horizontal, as exemplified by the photos above, or frontal, or sagittal. During familiarization, each subject individually used a physical device consisting in an aluminium hoop (1.8 m in diameter) that was materializing the plane of the MR to be performed during the experiment. Each subject memorized the four objects fixed by Velcro® scratches (a yellow disk, a red triangle, a blue square and a green star) and the respective locations of each object relative to him/her [(a.) with a subject standing in the centre of the hoop]. The subject was also trained to imagine which object would be at a predetermined location after a rotation (at 90° or at 180°), in one of the two possible directions in the rotation plane (*e.g.*: leftward or rightward in the horizontal plane) of either the hoop or the subject (a.). After removal of the objects from the hoop, the memorization of the objects and of their respective locations was verified and each subject was trained again to perform MR, as in the previous phase (b.). Thereafter, each subject realized a series of chronometric MRts, directly derived from this familiarization and which were shown on the screen of a laptop [(c.) showing another subject than a. and b. standing in front of the laptop].

During each phase of familiarization, each subject was initially placed in the middle of a physical device that consisted in a large aluminium hoop (Figure S3.a.). Depending on the phase of familiarization, the subject stood-up: (a) in front of the plane containing the hoop that was maintained vertically in a support, as close as possible to the hoop (in the F condition), (b) at the centre of the hoop that was placed horizontally to the ground (in the H condition), or (c) one foot on each side of the hoop that was maintained vertically (in the S condition). Four objects were initially placed on the hoop (Figure S3.a.) at the extremities of two orthogonal diameters of the hoop and the same object was systematically placed, in the F, H and S conditions, respectively: (a) at the feet of the subject, (b) in front of the toes of the subject, and (c) between the feet of the subject. Therefore, relative to the subject, the position of each object on the hoop was easy to define and unambiguous. For example, in the H condition, the four objects were respectively placed ~90cm from: (a) the subject's toes, (b) the subject's heels, (c) the subject's right ankle and (d) the subject's left ankle.

The subjects were asked to determine the object that would be at a predefined place relative to the avatar, after a given MR. Different predefined locations were used, *e.g.*: above the head or at the feet of the subject in F condition[6]. In each plane condition, each subject was trained to imagine eight different rotations, randomly experimented [*i.e.*: (two d° conditions) × (two Dir conditions) × (two FR conditions)]. The objects were initially placed on the hoop (Figure S3.a.) and then they were removed and imagined by the subject (Figure S3.b.) After familiarization with the MRt in one of the three Plane conditions (H, F, or S), the subject realized the experimental series directly derived from this experience (Figure S3.c.).

Such sequence was reiterated three times, *i.e.*: in each Plane condition. The order of the sequences was randomly assigned. Two consecutive sequences of familiarization and MRt series were separated by a five mn rest.

References

1. Bretz, F. Hothorn, T. & Westfall, P. *Multiple Comparisons Using R*. (Chapman and Hall/CRC Press, 2010).
2. Fox, J. & Weisberg, S. *An R Companion to Applied Regression.* (Thousand Oaks, CA: SAGE Publications, 2019).
3. Faraway, J. J. *Extending the linear model with R: generalized linear, mixed effects and nonparametric regression models*. (Chapman and Hall/CRC Press, 2016).
4. McCullagh, P. & Nelder, J. A. *Generalized linear models*. (Chapman and Hall/CRC Press, 1989).
5. Fox, J. & Hong, J. Effect displays in R for multinomial and proportional-odds logit models: Extensions to the effects package. *J. Stat. Softw.* **32**, 1-24, doi: 10.18637/jss.v032.i01 (2009).
6. Carpenter, M. & Proffitt, D. R. Comparing viewer and array mental rotations in different planes. *Mem. Cogn.* **29**, 441-448, https://doi.org/10.3758/BF03196395 (2001).
